# Advances and Breakthroughs in IRES-Directed Translation and Replication of Picornaviruses

**DOI:** 10.1128/mbio.00358-23

**Published:** 2023-03-20

**Authors:** Sahibzada Waheed Abdullah, Jin’en Wu, Xuefei Wang, Huichen Guo, Shiqi Sun

**Affiliations:** a State Key Laboratory of Veterinary Etiological Biology, College of Veterinary Medicine, Lanzhou University, Lanzhou Veterinary Research Institute, Chinese Academy of Agricultural Sciences, Lanzhou, China; Albert Einstein College of Medicine

**Keywords:** IRES, virus replication, ITAFs, translation, *Picornaviridae*

## Abstract

Viruses lack the properties to replicate independently due to the limited resources encoded in their genome; therefore, they hijack the host cell machinery to replicate and survive. Picornaviruses get the prerequisite for effective protein synthesis through specific sequences known as internal ribosome entry sites (IRESs). In the past 2 decades, significant progress has been made in identifying different types of IRESs in picornaviruses. This review will discuss the past and current findings related to the five different types of IRESs and various internal ribosome entry site *trans*-acting factors (ITAFs) that either promote or suppress picornavirus translation and replication. Some IRESs are inefficient and thus require ITAFs. To achieve their full efficiency, they recruit various ITAFs, which enable them to translate more effectively and efficiently, except type IV IRES, which does not require any ITAFs. Although there are two kinds of ITAFs, one promotes viral IRES-dependent translation, and the second type restricts. Picornaviruses IRESs are classified into five types based on their use of sequence, ITAFs, and initiation factors. Some ITAFs regulate IRES activity by localizing to the viral replication factories in the cytoplasm. Also, some drugs, chemicals, and herbal extracts also regulate viral IRES-dependent translation and replication. Altogether, this review will elaborate on our understanding of the past and recent advancements in the IRES-dependent translation and replication of picornaviruses.

## INTRODUCTION

The protein synthesis process in eukaryotes is accomplished in four phases, initiation, elongation, termination, and recycling. Translation initiates cap dependence during normal conditions and internal ribosome entry sites (IRESs) dependent in adverse conditions. Approximately 3.4 decades ago, IRES was discovered in picornaviruses, and shortly after this discovery, it was also identified in cellular mRNAs (1). IRES is a well-structured body composed of domains; every domain has its function, and collectively, they work as a team to facilitate viral translation initiation. Also, it has distinct secondary and tertiary structures that allow it to operate via numerous RNA-RNA and RNA-protein interactions essential for ribosome assembly (2). During viral infections and cellular stress, global protein translation is halted by various mechanisms. Viruses and some cellular mRNAs responsible for stress protein response utilize IRESs to alternatively carry on their protein translation during the adverse cellular environment (3). IRES activity may be supported by IRES *trans*-acting factors (ITAFs), which can stabilize a certain IRES conformation, enabling the small ribosomal subunit to attach directly to the mRNA (4). In addition to IRES-promoting or -suppressing activity, ITAFs are involved in many other physiological processes, such as invasion regulation and migration, cell differentiation and proliferation, progression of the cell cycle, and apoptosis (5, 6).

**FIG 1:**
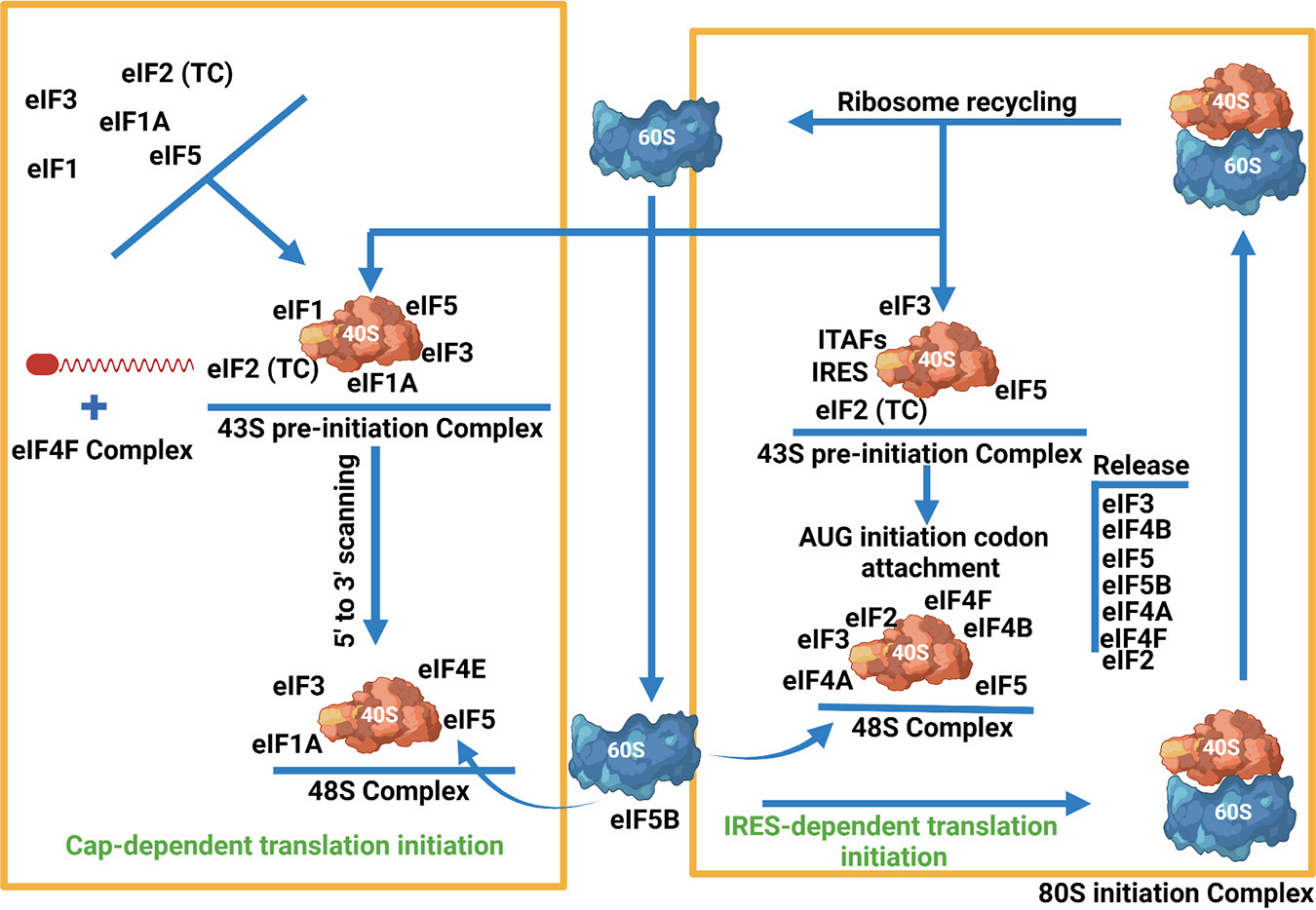
Protein synthesis in eukaryotic cells. In eukaryotic cells, protein synthesis initiation can occur through two mechanisms. Under normal conditions, cap-dependent translation initiation involves reconstituting eukaryotic initiation factors, including eIF1A, eIF1, eIF2, eIF3, and eIF5, with the 40S ribosomal subunit and Met-tRNAi to form the 43S preinitiation complex. tRNA scans the ribosome for the initiation codon, which is facilitated by the eIF4A, eIF1, eIF2, and eIF4F complex-forming 48S complex. At this stage, the 60S ribosomal subunit is recruited with the help of eIF5B and constitutes the 80S complex. Here, all the initiation factors are released, and the elongation step progresses. The 80S complex is then recycled for another translation initiation. In the case of cap-independent translation, not all the initiation factors are required to form a 43S preinitiation complex; in addition, ITAFs have a significant role in recruiting ribosomal subunits. Reconstitution of 48S complex requires eIF2, eIF3, eIF4A, eIF4B, eIF5, and eIF4F. The figure shows a general IRES-dependent translation initiation process.

The 5′ cap and poly(A) tail of the eukaryotic mRNA have an essential role in translational control, and together with cellular proteins and initiation factors, they are more potent by exerting a synergistic effect on cellular translation (7, 8). During normal conditions, translation initiation goes through a series of events; eukaryotic initiation factors (eIFs) facilitate the recruitment of ribosomes to the mRNA, and initiator tRNA (Met-tRNAi) is brought to the P site of the ribosome’s 40S (9). The eIF4B and eIF4A factors and cap-binding complex (eIF4F) are essential for the attachment of 43S complexes (composed of the 40S, eIF2/GTP/Met-tRNAi, eIF1, eIF1A, eIF3, and eIF5) on the 5′ terminus of capped mRNA; however, to facilitate scanning of the ribosome to the start codon is not sufficient. The eIF1 ability is enhanced through eIF1A to detach the anomalously assembled complexes from mRNA, leading to the assembly of the 48S complex at the start codon. The association of the 60S subunit with 48S is triggered by eIF5B to make the 80S initiation complex and the simultaneous dislodgment of initiation factors (eIF2-GDP, eIF3, eIF1, eIF4A, eIF4B, eIF4G, and eIF5). The initiation process ends with the eIF5B GTPase activity, which induces hydrolysis and release of GDP-bound eIF5B and eIF1A from the elongation-competent 80S ribosome, followed by the termination and recycling of ribosome (9) (Fig. 1).

In IRES-dependent translation initiation, the 43S preinitiation complex is formed by recombining initiation factors (eIF4A, eIF3, eIF2) in a cap-independent manner. In addition, most viral IRESs recruit ITAFs to the 40S ribosomal subunit to enhance the initiation process (10). The formation of the 48S initiation complex commences by joining initiation factors (eIF2, eIF3, eIF4A, eIF4B, eIF5, and eIF4F), ITAFs bounded ternary complex Met-tRNAiMet-eIF2-GTP, and attachment of the IRES AUG initiation codon (10–12). Finally, the 80S initiation complex is formed by binding the 60S ribosomal subunit to the 48S complex with the help of eIF5B and eIF5 (13) (Fig. 1). Besides this widely existing model of translation initiation, some cellular mRNA, under stress conditions, adopt the cap-independent translation for the survival of the cells, and therefore, some viral mRNA also follow this strategy to start translation initiation with the help of IRES located in 5′ untranslated regions (5′ UTRs) of the viruses (14). The limited resources of viruses compel them to opt for the host cellular machinery to translate their proteins and replicate. Some RNA viruses utilize IRES-dependent translation, hijacking various host proteins and recruiting ribosomes to satisfy the need for protein production and replication (15).

Various cellular proteins are RNA-binding proteins on which viral IRESs anchor to promote their translation initiation. This review focuses on the different host RNA-binding proteins and how they help or oppose a variety of picornaviruses during cell infection. We will shed light on the ITAF discoveries and some important factors through time and their important functions in IRES modulation of the *Picornaviridae*, making the translation more flexible and easier. Additionally, the importance of chemical drugs and herbal extracts in IRES-driven translation will be discussed. Finally, the host factors’ translocation to viral replication factories in the cytoplasm will be discussed.

## PICORNAVIRIDAE

The order *Picornavirales* consists of five families, *Picornaviridae*, *Iflaviridae*, *Secoviridae*, *Dicistroviridae*, and *Marnaviridae* (16). *Picornaviridae* is divided into 68 genera and 158 species (17, 18). The viruses of this family can infect a wide range of hosts, such as birds, mammals, fish, amphibians, and reptiles. Numerous picornaviruses are of significant human and veterinary importance, which may cause illnesses of the upper respiratory tract, central nervous system, liver, heart, skin, and gastrointestinal tract (19). Below, we will discuss various viruses of the *Picornaviridae* that cause diseases in different hosts and will try to highlight their significance and challenges to future research. Poliomyelitis is a lethal human disease affecting the central nervous system with temporary or permanent paralysis. It is caused by poliovirus (PV), and the recent reemergence in New York is alarming (20). Human rhinovirus type 2 (HRV-2) infection in immunocompetent hosts may be minor and self-limiting; nevertheless, it may also be linked to pneumonia in immunosuppressed individuals, bronchiolitis in neonates, and exacerbations of preexisting respiratory diseases such asthma or chronic obstructive pulmonary disease. The unavailability of a licensed vaccine is another challenge to researchers (21).

Hand-foot-and-mouth disease (HFMD) and herpangina are caused by enterovirus A71 (EV-A71). HFMD is a highly contagious and life-threatening disease, and there is an urgent need for a vaccine to eradicate this disease (22). Apart from human viruses, foot-and-mouth disease virus (FMDV) causes a highly contagious and economically important disease in cloven-hoofed animals. Although some vaccines are available, this disease has yet to be eradicated from most parts of the world (23). Encephalomyocarditis virus (EMCV) may cause sudden death due to myocarditis at any stage of life in pigs, and reproductive disorders, including abortion, stillbirth, and mummification of the fetus, can be observed in sows. Until now, no commercial vaccine has been available to combat the disease (24). Hepatitis A virus (HAV) infects approximately 1.5 million people annually, usually affecting children. The incidence is high in developing countries. The virus mainly proliferates in the hepatocytes. Patients develop pale stool and dark urine followed by jaundice, pruritis, and icteric sclera. HAV infection is not a serious problem and is often a self-recovering disease, but supportive therapy is required during the disease (25). Acute gastroenteritis in humans caused by the Aichi virus (AV) and the seroprevalence data collected from Japan, Tunisia, France, Spain, and Germany showed that 80 to 99% of adults have antibodies, indicating the high incidence and exposure of the population to the virus (26). Picornaviruses and the diseases caused by them described above poses important public and animal health threat. These viruses should be thoroughly studied to find out the remedies to improve animal and public health.

The distinguishing characteristics of this family include very small viruses ranging from 22 to 30 nm, with no envelope or capsid surrounding the positive-sense single-stranded RNA having a single open reading frame (ORF), with some exceptions, such as the viruses of the genus *Dicipivirus* (27, 28). Most picornaviruses have similar genome structures composed of L, 2A, 2B, 2C, 3A, 3C, 3D, 3′ UTR with a poly(A) tail, and VPg proteins. VPg, also known as 3B, serves as a primer for FMDV replication and is linked to the 5′ UTR (19). The capsid comprises four capsid proteins, 60 copies each of identical particles known as protomer, VP1, VP2, VP3, and VP4. The genomes of picornaviruses contain 5′ UTRs, which possess crucial and highly structured stem-loops known as IRESs. IRESs assemble the ribosomes and facilitate the cap-independent translation (29). The cap-independent translation in the picornaviruses family is mainly regulated by ITAFs. These ITAFs bind to various IRES regions to initiate the translation. However, ITAFs may positively or negatively regulate IRES activity (30, 31).

### IRES structures in *Picornaviridae*.

IRES was first described in PV and EMCV (1). However, IRES has been reported to exist in cellular mRNAs (32). We will focus on the viral IRESs in this review, specifically *Picornaviridae*. There are five types of IRESs found in picornaviruses, classified as follows. (i) They may have similar structures and sequence motifs (2, 33). Every IRES type adopts a different mechanism to manipulate the 40S subunit to the start codon. (ii) These different types of IRES mechanisms rely on the unique interaction of the cap-dependent component (eIF4E) of the translation machinery with the cap-independent components of the IRES (34). (iii) The ITAF requirement is another method of IRES classification, where IRESs hijack or require ITAFs to promote IRES-dependent translation initiation. Below, we will briefly introduce the five types of IRESs classified in *Picornaviridae*. Type I IRES is found in poliovirus, human rhinovirus type 2, and EV-A71. Type I IRES-containing viruses utilize a ribosomal scanning mechanism for start codon recognition. This process is assisted by various initiation factors and host cellular ITAFs (15, 35). FMDV and EMCV contain type II IRESs, where two start codons are present at the 3′ end of the FMDV IRES. The ribosome is directly recruited to the only start codon in the case of EMCV. In the case of FMDV, the ribosome is recruited with the help of a second start codon, assisted by various initiation factors and ITAFs (35, 36). Type III IRES is present in the hepatitis A virus. Translation initiation requires initiation factors and ITAFs. In contrast to other IRES types, type III IRES of the hepatitis A virus requires intact eIF4E (37, 38). Type IV IRES was discovered in flaviviruses and later reported in picornaviruses in senecavirus A (SVA). The unique characteristic of this type of IRES is that it does not require any initiation factors and ITAFs to initiate its translation (39, 40). Type V IRES is found in Aichi virus A of the *Picornaviridae*, and in some viruses, the effective translation initiation requires the DExH-box protein DHX29 (41, 42). Models of all IRES types have been proposed, but only a few structures have been experimentally determined. Significant differences exist among them based on RNA secondary structures and RNA-binding protein requirements. However, natural selection makes these viruses evolve their IRES three-dimensional structures to suitably fit the environment of the host cell and make their proteins without any hindrance. Here, we will discuss the five IRES types and the latest development in IRES-dependent translation.

## TYPE I IRES AND THE ITAFS REGULATING ITS TRANSLATION

### Type I IRES structure.

Type I IRES is found in poliovirus, HRV-2, EV-A71, bovine enterovirus (BEV), and coxsackievirus type B3 (CVB3) (43–45). The 5′ UTR of poliovirus is approximately 743 nucleotides (nt) long and is divided into six domains, I to VI, and a pyrimidine tract (Yn) of 8 to 10 nt, separated by a spacer (Xm) of 18 to 20 nt from an AUG triplet (Yn-Xm-AUG motif) at its 3′ border. This motif is believed to be the entrance site of ribosomes into the 5′ UTR. The initiation codon for the polyprotein lies at a variable distance downstream of the 3′ border of the IRES (40, 46). The clover leaflike structured domain I function is to facilitate viral negative- and positive-strand replication (40). It is also responsible for preventing the PV RNA from degradation by cellular nucleases (47). Domain II is called stem-loop (SL) II and significantly regulates IRES translation via ITAFs (48). Domain III is required for the IRES activity and is responsible for interacting with ITAFs (35). Domain IV of the poliovirus has various important regions, including the C-rich motifs, which are critically required for binding to the *trans*-acting factor poly(rC)-binding protein 2 (PCBP2) (49).

**FIG 2:**
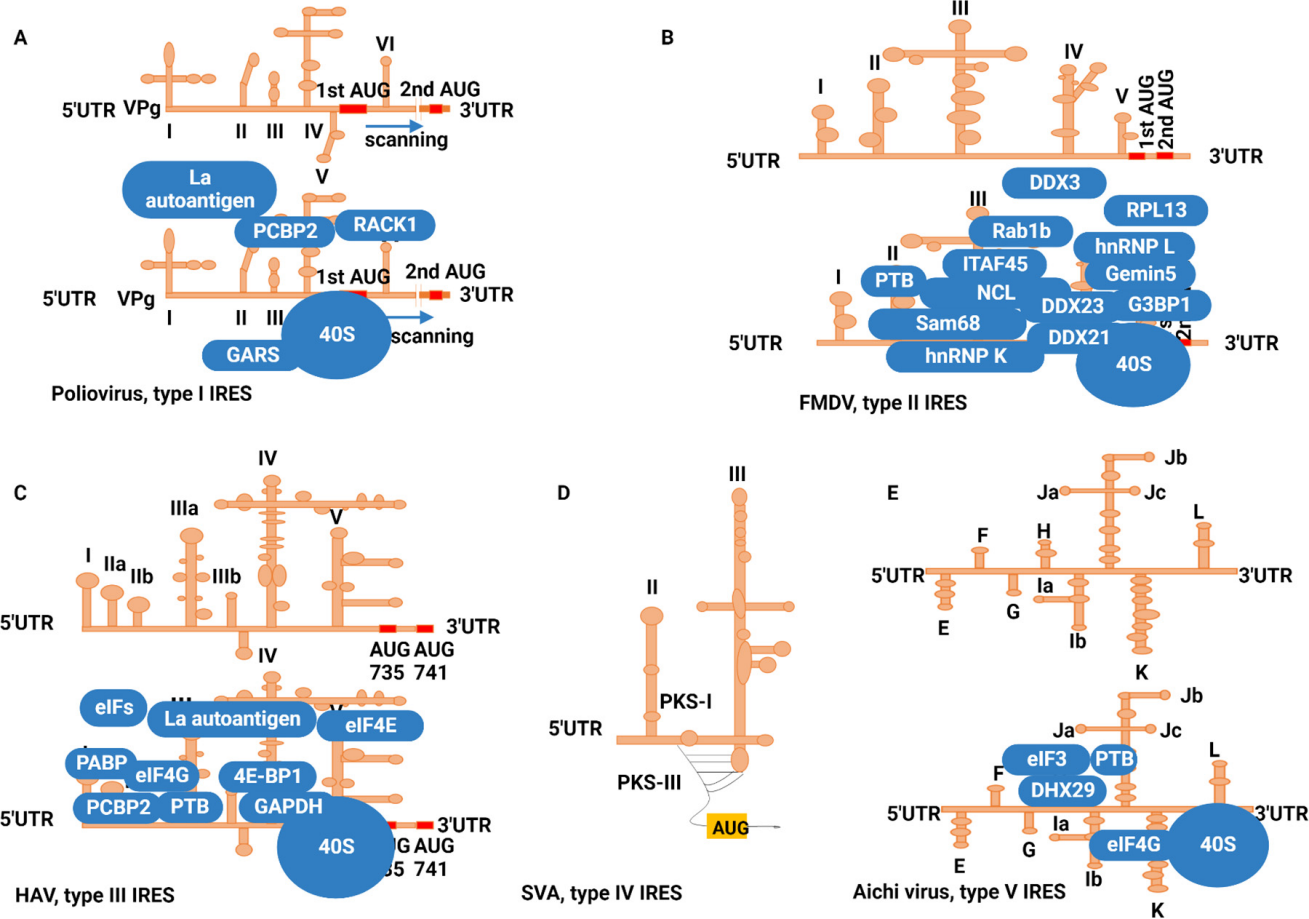
Five types of IRESs in *Picornaviridae*. (A) Poliovirus contains type I IRES, which requires all initiation factors for translation initiation. Type I IRES anchors many ITAFs to its various domains for efficient translation. (B) Schematic diagram of type II IRES found in FMDV. There are five domains in FDMV IRES; domain III is the main domain that hijacks various ITAFs for translation initiation. (C) Type III IRES is only found in HAV of *Picornaviridae*. HAV IRES has been considered inefficient; therefore, it associates with ITAFs to enhance its translation needs. (D) Type IV IRES is found in senecavirus A and non-*Picornaviridae* CSFV. This type of IRES does not require ITAFs for translation initiation. (E) Type V IRES is found in Aichi virus, which contains eight domains. The canonical initiation factors are insufficient for translation initiation, where PTB is a significantly crucial ITAF in AV IRES activity.

A recent report has shown the existence of variability in the 5′-UTR sequences of different lineages of EV-D68. The phylogenetic study showed that there were greater genomic diversities in the 5′ part of the 5′ UTR rather than the 3′ part of the 5′ UTR of the IRES of EV-D68. The study further indicated three lineages of EV-D68; however, lineage 2 had the greatest IRES activity due to the mutation in stem-loop IV (50). This report indicated that IRES sequences of different lineages of the same virus should be strictly monitored to keep vigilance on virus replication behavior. The next important region is GNRA, which is required for tertiary interactions (51). Domain V and VI stimulate the 43S ribosomal preinitiation complex binding, which continues scanning until the initiation codon (43). The schematic diagram of the type I IRES is shown in Fig. 2A.

### Initiation factor requirement.

Several IRES types are inefficient, and initiation factors play critical roles in IRES-dependent translation initiation for the complete exploitation of IRES efficiency. Although some IRES types do not strictly depend on initiation factors, type I IRESs require all eIFs for efficient translation initiation except eIF4E (40). ITAFs and several adopter molecules like eIF4G, responsible for functional circularization and increase of protein synthesis efficiency, are required to regulate type I IRES activity (43, 52).

### Positively regulating ITAFs.

Many ITAFs bind to different regions and domains of type I IRES to stimulate or inhibit IRES activity (Table 1), whereas IRES domains are highly significant regions in translation initiation. The pioneering ITAF of poliovirus was described earlier in 1989 and named La autoantigen. It was shown that La autoantigen promoted the poliovirus mRNA translation in HeLa cells, and its addition to rabbit reticulocyte lysate (RRL) (translation of poliovirus mRNA is inefficient in RRL) also increased viral mRNA translation. In addition, viral infection induced nuclear protein La autoantigen translocation to the cytoplasm (53). These findings triggered researchers to identify proteins involved in viral translation initiation. Below, we will discuss various viral strategies and try to understand how different viruses harness host ITAFs to promote their viral translation initiation. The poliovirus ITAF, PCBP2, contains multiple RNA-binding domains, each of which engages with a different site on the IRES and thus restricts its conformational flexibility and helps enforce its adoption of a specific conformation (49). It was shown that the apical region of poliovirus SLIV, known as GNRA, was not crucial for binding to the PCBP2; however, its mutated form has shown adverse effects on the infectivity of poliovirus (49). PCBP2 can bind to domain IV, and clover leaflike structure domain I is thus considered important for the IRES activity of most enteroviruses. In contrast, it has no role in type II-containing IRES activity of EMCV and FMDV (54).

**TABLE 1:** ITAFs, eIFs, and other factors required for the translation initiation of IRESs

IRES type	Virus	ITAF, eIF, or other factors required	Positive or negative regulator^*a*^	Interaction regions in IRES	Reference(s)
Type I IRES	Poliovirus	La autoantigen	+	Not determined	53
		PCBP2	+	Domain IV and I	49
		GARS	+	Domain V	56
		RACK1	+	Not determined	57
	Human rhinovirus type 2	Unr	+	Domain II, V, and VI	58
		PTB	+	Not determined	58
	Enterovirus A71	hnRNP K	+	Stem-loop I and II	61
		FBP1	+	Linker region (636–745)	62
		DDX3	+	IRES + spacer, domains I and II, V and VI, and VI + spacer	63
		Ago2	+	Stem-loop II	64
		Hur	+	Stem-loop II	64
		hnRNP A1	+	Stem-loop II (bulge 5′-AYAGY-3′) and hairpin loop 5′-RY[U/A]CCA-3′	48
		Staufen1	+	5′ UTR	66
		PTB	+	Stem-loop VI	67
		HSPA6	+	Not determined	69
		FBP2	−	Stem-loop I and II, II and III, VI + spacer	73
		AUF1	−	Stem-loop II	76
	Coxsackievirus type B3	PSF	+	Stem-loops I, IV, and V	70
		La autoantigen	+	5′ UTR	71
Type II IRES	Foot-and-mouth disease virus	PTB	+	Domain II	102
		ITAF45	+	Central domain (domain III)	11
		Sam68	+	Domains II–IV and IV	104
		Rab 1b	+	Domain III	106
		DDX3	+	IRES (no specified domain determined)	107
		NCL	+	Domains III, IV, and V	108
		Gemin5	−	Domain V	111
		G3BP1	−	Domain V	116
		hnRNP K	−	Domain II, III, and IV	118
		hnRNP L	−	Domains IV and V	119
		DDX23	−	Domain III and IV	120
		DDX21	−	Domains I and II, III, and IV	121
	Encephalomyocarditis virus	eIF4G, eIF3, eIF4A, and eIF4B	+	Upstream of the initiation codon	124
		PTB	−	Binds at various domains	127
		RACK1	−	Not determined	57
		DDX60	−	Not determined	129
Type III IRES	Hepatitis A virus	PCBP2	+	The start of HAV IRES (1–157)	135
		PTB	+	Domain IIIa	30, 31
		eIF4G	+	Not determined	136
		eIF4E	+	Not determined	134
		PABP	−	pY1	137
		La autoantigen	−	Domain IIIa	30
		GAPDH	−	Domain IIIa	30, 138, 177
		4E-BP1	−	Not determined	38
Type V IRES	Aichi virus	eIF4G	−	Domain K	152

a +, positive; −, negative.

Furthermore, there are three hypothetical scenarios under which PCBP2 promotes type I IRES activity. First, the association between type I IRES and PCBP2 directly recruits the ribosome through stabilizing RNA secondary and tertiary structures. Second, the interaction of PCBP2 with the type I IRES is directly associated with the ribosome and promotes translation. Third, the translation factors may be recruited by the interaction of IRES and PCBP2 via protein-protein interaction or direct recruitment of other proteins to RNA (54, 55). Glycyl-tRNA synthetase (GARS) is an important component of PV RNA translation. It interacts at the apical region of domain V near the binding site of eIF4G of the poliovirus IRES. The binding of GARS enhances the accommodation of the initiation region of the IRES in the mRNA-binding site of the ribosome, hence significantly boosting the activity of the IRES during the 48S initiation complex formation (56). A recent study showed that overexpression of RACK1 enhanced poliovirus IRES luciferase activity. Furthermore, cells lacking RACK1 showed decreased viral titer and plaque size, indicating a low level of 2A and 3C proteases. Low levels of proteases are unable to cleave more host translation initiation factors, eIF4G, and poly(A)-binding protein (PABP), leading to a delayed host translation shutoff and decreased viral IRES activity (57); however, it was not shown whether the positive regulation of PV IRES activity was due to the direct interaction of viral IRES with the RACK1.

The Unr binds to domain II and the top region of domains V and VI of the HRV-2 IRES, and it also maintains the complex tertiary structure to ensure the translation effectively. Similarly, mutations in the HRV-2 subdomain II and V showed decreased translation stimulation by polypyrimidine tract-binding protein (PTB), indicating these domains’ significance for PTB-dependent IRES activity. Furthermore, PTB showed a synergistic effect with the Unr in HRV-2 IRES translation (58, 59).

In EV-A71 IRES, ribosomes first attach upstream of the start codon and then scan the mRNA until an appropriate downstream AUG initiation codon comes across and protein synthesis begins (60). Heterogeneous nuclear ribonucleoprotein K (hnRNP K) was reported as an EV-A71 IRES stimulatory ITAF, which translocated to the cytoplasm and interacted with viral 5′-UTR IRES to promote viral production and replication. The viral loads were significantly lower in hnRNP K-knockdown cells than in mock cells. Furthermore, the hnRNP K, KH, proline-rich domain, and KH2 interacted with the EV-A71 5′-UTR IRES stem-loops I and II. However, the proline-rich domain is the key player in hnRNP K interaction with other cellular proteins (61).

The nuclear protein, far upstream element-binding protein 1 (FBP1), binds with the linker region in IRES of EV-A71 (636 to 745 nt) and positively regulates the viral IRES activity. During the viral infection process, FBP1 was translocated into the cytoplasm. The purpose of translocation to the cytoplasm was to compete with negative IRES regulatory ITAFs to enhance viral replication. Protein cleavage by viral proteases is a common phenomenon usually observed during viral replication. The 2A proteinase of EV-A71 cleaved the FBP1 at the Gly-371 residue during the infection process, producing a functional product that upregulates the viral yield (62).

Similarly, EV-A71 viral proteases 3C^pro^ and 2A^pro^ cleaved eIF4G to inhibit host translation and promote viral replication. DDX3 interacted with EV-A71 IRES at various regions like IRES plus spacer, domains I to III, domains IV to VI, and domain VI plus spacer without any preference, although truncated eIF4G preferred to attach to viral IRES by interacting with IRES plus spacer and domains IV to VI. DDX3 interacts with EV-A71 IRES inefficiently, but it destabilizes the IRES domain VI structure, which helps its initiation codon AUG to be tracked by the 43S ribosomal complex and promotes IRES activity (63). Protein-protein interaction is an important phenomenon that may have peculiar effects on virus replication. Ago2 and Hur interacted with the EV-A71 IRES stem-loop II to enhance viral translation. When Ago2 and Hur were simultaneously knocked down, a millionfold decrease in EV-A71 viral IRES activity and replication was observed. In contrast, the knockdown of either protein decreases the viral yield 1,000-fold, suggesting that these two proteins pose a synergistic effect to regulate IRES-driven translation positively (64).

Correspondingly, the UP1 domain of the hnRNP A1 interacted with EV-A71 IRES stem-loop II through bulge 5′-AYAGY-3′ and hairpin 5′-RY[U/A]CCA-3′, which positively regulate viral IRES activity. Furthermore, the stem-loop II was significantly important, associated with small viral-derived RNAs and four other proteins. Small viral-derived RNAs are effective regulators of host gene expression during virus-host interactions. In contrast, mutation studies indicated that the regions in the stem-loop impair viral replication and thus are considered a potential antiviral target (48, 65). Likewise, Staufen1, an RNA-binding protein, interacted with the 5′-UTR IRES of the EV-A71 virus through the RBD2-3 domain and enhanced its translation and replication. Moreover, the binding site in Staufen1 could be associated with other cellular proteins, which could indirectly help the EV-A71 IRES activity; furthermore, the polysome fractionation assay confirmed that it influences the RNA stability of the EV-A71 5′ UTR (66).

Nuclear polypyrimidine tract-binding protein has been reported to play a crucial role in the IRES activity of some viruses. It enhances the IRES activity of EV-A71 by binding through RNA recognition motif (RRM) 1 and 2 to the stem-loop VI of the IRES (67). In contrast, PTB’s RRM 1 and 2 interact with the basal part of domain V of poliovirus IRES by regulating the eIF4G interaction. The difference in attachment sites might be due to different viruses, although it enhanced the viral IRES activity (68). Another example of the diverse mechanisms utilized by viruses to hijack proteins and harness them for their replication is that HSPA6 promoted EV-A71 IRES activity via chaperoning cellular proteins instead of viral proteins (69). PTB-associated splicing factor (PSF) interacts with both the IRES and the cloverleaf RNA and is essential for viral RNA translation. PSF knockdown significantly decreased luciferase activity. The CVB3 replicon transfection and infection significantly increased PSF protein and mRNA levels. This indicated that CVB3 promotes PSF protein expression and mRNA level to enhance its IERS translation. It was shown that PSF strongly interacts with CVB3 full-length IRES and cloverleaf RNA SL-1, whereas moderate interaction was observed with SL-4 and SL-5 (70). CVB3 adopted a similar phenomenon to hijack the La autoantigen to promote its IRES-dependent translation. During CVB3 infection, pancreatic tissue is the main target for the virus to proliferate, and an abundant amount of La autoantigen protein was detected. The La autoantigen showed interaction with the 5′ UTR of the CVB3 (71). We concluded from the above-described studies that viruses use the following strategies to hijack host proteins and utilize them for translation initiation: (i) interaction with ITAFs through specific sequences in viral IRESs to induce conformational changes necessary for efficient translation initiation, (ii) protein-protein interaction, which may have a synergistic effect on viral IRES activity, (iii) migration of ITAFs by viruses, and (iv) cleavage induced by viral proteases.

### Negatively regulating ITAFs.

Some proteins negatively regulate viral translation and replication, as shown in Table 1. Far-upstream element-binding protein 2 (FBP2) interacted with stem-loops I and II, II and III, and VI plus the spacer region of the EV-A71. It was shown that FBP2 negatively regulated EV-A71 IRES activity (72). However, during EV-A71 infection, FBP2 was hijacked and cleaved by viral protease and further degraded via the proteasome, caspase, and autophagy pathways. Interestingly, upon the C-terminal cleavage of FBP2, it altered its negative IRES regulatory activity to positive (73). Recent studies show that AU-rich element-binding factor 1 (AUF1), a nuclear resident localized to the cytoplasm with EV-A71 infection, interacted with the stem-loop II domain of the type I IRES of EV-A71 and negatively regulated its translation. However, this negative regulation was due to the competitive binding of AUF1 and hnRNP A1 to the stem-loop II (74–76). In addition, mutating the central bulge sequence, 5′-UAG-3′ to 5′-CCC-3′ of the SLII, inhibited the viral replication by changing the conformational orientation (48). More ITAF discoveries that negatively regulate type I IRES are warranted to better understand virus-host interaction mechanisms.

### Herbal extracts and chemicals and drugs influencing type I IRES activity and virus replication.

Herbal extracts and some chemicals or drugs showed inhibitory effects on IRES-driven translation. The chemical kaempferol, a flavonoid, interfered with the EV-A71 viral IRES with the help of *trans*-acting factors HNRH1, HNRPD, HNRPF, and FUBP1, which progressively increased cell viability and inversely decreased viral translation and replication (77). Similarly, the herbal extract geniposide decreased the EV-A71 and encephalomyocarditis virus replication as well as the IRES activity by 50 to 80%, whereas a 40 to 60% reduction was observed when treated with Fructus gardeniae, which indicated the efficacy of these herbal extracts to inhibit type I and type II IRES activity (78). These chemical compounds may interfere with the interaction between viral ITAFs and IRESs. For example, idarubicin (IDR) is an anthracycline anticancer compound that dramatically blocks viral RNA and protein synthesis, albeit it does not affect the proteolysis process of the virus. Furthermore, IDR-dependent inhibition of EV-A71 IRES translation was correlated with the binding affinity between IRES and the IDR. The IDR-induced inhibition was accomplished through binding between the *trans*-acting factor of the host, hnRNP A1, and EV-A71 IRES (79).

The medicinal plant Peganum harmala is a rich source of a beta-carboline alkaloid, known as harmine, which inhibits the NF-κB pathway and EV-A71 replication and plague formation during viral infection. In addition, it significantly decreased the viral IRES activity in a dose-dependent manner (80). Recently, another plant extract, a flavonoid, prunin, a narrow-spectrum antiviral agent against EV-A71, suppressed the viral PFU, titer, and viral RNA and disrupted protein synthesis. The prunin-resistant EV-A71 mutant was obtained by consistent prunin supplementation. The sequence data confirmed the five base pairs substituting stem-loops II and IV and connecting the stem-loop between II and III. Furthermore, dual-luciferase assay and plaque reaction assay revealed that the substitution in this area of IRES resulted in the same resistance as prunin, suggesting the importance of stem-loop II EV-A71 IRES in prunin resistance (81).

Expanding the body of knowledge regarding natural compounds which affect the translation and replication of type I IRES viruses, Tang et al. showed that the antiprotozoal drug emitine has antiviral effects against EV-A71 by suppressing IRES-driven translation. Furthermore, this suppression was independent of various cell types, such as Vero cells, 293T cells, and rhabdomyosarcoma (RD) cells (82). The studies described above suggested that herbal extracts and chemical drugs are important in suppressing type I IRES activity. The data presented in this review regarding herbal extracts affecting IRES-dependent translation showed their importance and significance; however, more research is needed to find more potent remedies against this group of viruses. Future studies should focus on the discovery of novel herbal extracts and chemical drugs causing interference between viral ITAFs and IRESs to limit viral replication.

## TYPE II IRES AND THE ITAFS REGULATING ITS TRANSLATION

### Type II IRES structure.

The genera *Aphthovirus* and *Cardiovirus* of *Picornaviridae* contain type II IRES. The genus *Aphthovirus* is mainly composed of animal viruses, bovine rhinitis A viruses 1 and 2; bovine rhinitis B viruses 1, 2, 3, 4, and 5; equine rhinitis A virus; and FMDV (83). *Cardiovirus* comprises six species, *Cardiovirus A*, *B*, *C*, *D*, *E*, and *F* (84). Type II IRES drives translation initiation in FMDV; the Lb and Lab leader proteinases are induced by incorporating two AUG in-frame codons (85).

The type II IRES of FMDV and encephalomyocarditis virus is located in the 5′ UTR of the genome. It is approximately 450 nt long and composed of five domains. Domain I is mainly involved in virus replication (86). The pyrimidine tract sequence in domain II of FMDV and EMCV functions to recruit the pyrimidine-binding protein (87). In contrast, domain III is crucial for attachment with *trans*-acting factors and RNA-RNA interaction, facilitated by its long and flexible structure. However, certain loops like GNRA and RAAA are important for IRES activity (88). Domain IV of the EMCV is close to the Yn-Xm motif and eIF4G binding that is enhanced by binding of the eIF4A to initiate the translation (11, 89). However, any mutation to the motifs or stem can drastically affect the translation efficiency, indicating that the RNA structure is more preserved and cannot afford the mutation (90). The eIF4G/eIF4A makes conformational remodeling on the ribosome attachment sites, hence promoting the attachment of the IRES and 43S complex (91). The fifth domain of FMDV IRES contains a conserved hairpin loop and a polypyrimidine-rich sequence (92). It is believed to be significant in recognizing and starting viral protein synthesis.

Moreover, the mutation in this domain is highly lethal to IRES activity (93). In the 3′ end of the IRES containing Yn-Xm motif, the 20-nt Xm spacer separates the pyrimidine-rich tract from the AUG start codon. Furthermore, the two initiation codons in FMDV are separated by 84 nt from each other, Lab AUG and Lb AUG; however, the second initiation codon is usually used for translation initiation. The schematic diagram of the type II IRES is shown in Fig. 2B. Mutating the 2nd AUG codon affected the replication of the FMDV; nevertheless, it did not affect the viability of the virus. The decrease in replication was due to the mutated 2nd Lb AUG codon, which consequently influenced the L protein's ability to cleave the eIF4G1 and inhibition of interferon (IFN) expression (94). Both of the initiation codons can start translation initiation in FMDV, while in the case of EMCV, initiation occurs at the equivalent of AUG-1, which is part of the Yn-Xm-AUG motif (95–97).

Furthermore, FMDV has extra SLs, SL-1, SL-2, SL-3a, and SL-3b; they are critical for IRES activity, and the absence of these SLs severely affects viral IRES activity (98). However, its activity depends on the 3′ UTR and is enhanced by various host proteins. Using a chimeric bicistronic construct containing 3′ UTR and IRES transfected into the cells, analysis of the bicistronic RNA level showed an overwhelming trigger by the 3′ UTR (99). The Martinez-Salas group introduced a point mutation in the IRES of FMDV; the translation activity was enhanced from 1.5- to 5-fold in mutated FMDV IRES compared to nonmutated (100). Type II IRES requires ITAFs, which either enhance or repress the IRES activity, just like type I IRES.

### Positively regulating ITAFs.

This section will discuss the ITAFs that promote the FMDV IRES activity, as listed in Table 1. A crucial ITAF, PTB, is a part of the 80S and 48S ribosomal initiation complex during the FMDV internal ribosome entry site RNA recruitment of the ribosome. It promoted the FMDV IRES translation efficiency by making the PTB-IRES complex and joining with small ribosomal subunits (101). The above-described study could not confirm the exact interaction site in FMDV IRES; however, another study showed that PTB interacted with domain II of the FMDV IRES and worked as an RNA chaperone to stabilize the viral IRES structure (102). Correspondingly, ITAF45, an important ITAF for FMDV, cooperates with PTB to promote FMDV IRES-dependent translation. ITAF45 interacts with the central domain (domain III) of the FMDV IRES (11). A Lys-rich motif in the C terminus of ITAF45 was the major RNA-binding site, facilitating the interaction with the FMDV IRES (103). Nuclear protein Sam68 interacts with the FMDV IRES at various domains. Its knockdown suppresses the viral IRES-driven translation, and a significant decrease in viral titer was observed. Moreover, it was confirmed that 3C^pro^ causes the cleavage of the Sam68 to facilitate viral replication (104). Furthermore, Sam68 interacted with domains II to IV and domain IV of the FMDV IRES. Sam68 binding affinity was decreased with domains IV and III by introducing a mutation in the motif RAAA, which resulted in nonviable viral progeny production. Besides, the Sam68-KH deletion mutant showed abolished ribonucleoprotein complex formation (RNP) (105).

The host proteins ARF5 and Rab1b interacted with domain III of the IRES. Interestingly, the IRES activity was diminished by ARF5, whereas Rab1b stimulated the IRES activity. The IRES-driven RNA showed localization with the Rab1b and ARF5, which indicated the localization of RNA to the endoplasmic reticulum (ER) Golgi components, indicating the role of the FMDV IRES domain III in localizing the RNA in the proximity of the ribosome-rich compartment (106). Protein-protein interactions are crucial for various cellular activities; thus, viruses benefit from them. A recent study showed that DDX3, in cooperation with RPL13, promoted FMDV IRES activity. This insight study showed that the knockdown of DDX3 interrupts the binding of RPL13 with FMDV IRES, and the knockdown of RPL13 affects the FMDV IRES-driven translation (107). Correspondingly, nuclear protein nucleolin (NCL) directly interacted with the FMDV IRES domains III, IV, and V and positively regulated FMDV IRES-driven translation. NCL directly enhanced the FMDV viral protein level but indirectly affected the FMDV mRNA level. Furthermore, NCL enhanced the translation initiation process by recruiting the translation initiation complexes to the viral IRES (108).

### Negatively regulating ITAFs.

On the other hand, some ITAFs downregulate FMDV IRES-dependent translation, as shown in Table 1. The ITAF Gemin5 was reported as a multitasking protein, involved in RNA splicing, snRNP biogenesis, nuclear and cellular mRNA recognition, and translation control (109, 110). A previous study showed that Gemin5 directly interacted with domain V of the FMDV IRES. The downregulation of Gemin5 increased, and overexpression decreased the FMDV IRES efficiency. Pulldown assay unveils that Gemin5 forms two complexes, eIF4E containing IRES-independent complex and ribonucleoprotein-IRES complex, which play a critical role in decreasing FMDV IRES-dependent translation (111). Further studies showed that Gemin5 was proteolyzed via L^pro^ of the FMDV into p57 and p85 products but not by EMCV (112). The C terminus of Gemin5 was responsible for interacting with the IRES (113). In contrast, Francisco-Velilla reported that the N terminus of Gemin5 binds directly to the ribosomal proteins L4, L3, and 60S ribosomal subunit, hence controlling global protein synthesis (114). The tetratricopeptide (TPR)-like domain in Gemin5 makes a dimer structure that anchors translational and splicing factors. In contrast, the mutation in this TPR dimer area was significantly lethal and abrogated translation promotion through p85 (115).

Similarly, a multifunctional protein residing in the stress granules (SGs), Ras GTPase SH3 domain-binding protein 1 (G3BP1), negatively regulated FMDV IRES translation. G3BP1 has shown an association with domain V of the FMDV IRES, PTBP, and translation initiation factor 4B (116). Like Gemin5, G3BP1 was cleaved into N-terminal G3BP1 and C-terminal G3BP1 by L^pro^ and subsequently suppressed the stress granule aggregation (117). Likewise, hnRNP K was reported as a negative regulator of FMDV IRES-driven translation. It binds to FMDV IRES domains II, III, and IV via its KH2 and KH3 domain sites and inhibits viral translation and replication. However, in counterattack, FMDV 3C proteinase cleaves hnRNPK to antagonize its effect. Interestingly, C-terminal hnRNP K 364 to 465 showed the opposite function to the full-length hnRNP K by promoting the viral mRNA and titer (118). Another member of the hnRNP family, hnRNP L, showed association through its RNA recognition motifs 3 to 4 with FMDV IRES domains IV to V and downregulated viral replication. Surprisingly, hnRNP L does not affect the IRES-dependent translation (119), as seen in hnRNP K (118). Recently, DDX23 was reported as a negative regulator of FMDV IRES translation and replication by interacting with viral IRES domains II, III, and IV; furthermore, FMDV infection degraded DDX23 via the lysosomal pathway, and similar to hnRNP K, FMDV 3C proteinase degraded DDX23 (120). Similarly, another member of the dead box helicase protein family, DDX21, negatively regulated FMDV viral replication and translation by interacting with viral IRES (121). The above-described studies showed one thing in common: FMDV hit back by cleaving or degrading host proteins to antagonize their antiviral activities.

### Positive and negative regulating ITAFs of EMCV.

The next important genus of the family *Picornaviridae* is *Cardiovirus*, which also contains type II IRES, where the L protein is present in its genome but lacks protease activity (19). The eIF4G binds upstream of the initiation codon with the EMCV mRNA IRES, leading to the recruitment of the viral mRNA to the ribosome for translation initiation via eIF3. The eIF4G in IRES-mediated translation initiation recruits eIF4A and 4B to the IRES, possibly as a prelude to the accommodation of the initiation codon and flanking regions in the mRNA-binding cleft of the 43S complex (122). IRES-mediated initiation in encephalomyocarditis virus is slightly different from FMDV, where initiation does not need eIF1A, eIF1, and the eIF4E subunit of eIF4F, and initiation does not need scanning (123). eIF4G and eIF4A promote the 43S complex association, the subunits of eIF4F that bind instantly upstream of the EMCV start codon (124). However, the Chamond group presented a contrasting model; the molecular mechanism of EMCV-IRES involves direct recruitment of the 40S subunit, suggesting that the viral IRESs are the primary ribosome binder, although the affinity of the EMCV IRES to the ribosomal 40S subunit is low and by itself is insufficient for the formation of a stable active initiation complex. The affinity of IRES for the 40S subunit may be involved in positioning it on the 40S ribosome after eIF4G/eIF4A-mediated recruitment (125).

The EMCV 5′ untranslated region promotes the internal initiation of translation with the help of an ITAF, PTB. In contrast, for other viruses, such as hepatitis C virus and Theiler's murine encephalomyelitis virus, internal translation initiation is unaffected by the PTB (126). It was sorted out that the PTB’s RBD3 domain interacted with the 5′ UTR through domain D with higher affinity than H, whereas RBD1 and RBD2 interacted near the 3′ UTR with EMCV-IRES F and K domain and thus stabilized and constrained the IRES 3D structural folds (127). A previous study showed that removing the hairpins ΔK2′ and ΔJ5 from the IRES of encephalomyocarditis virus mechanistically impaired IRES activity. In addition, binding with eIF4G/eIF4F was affected by the deletion of J and K domains, leading to reduced or no IRES-mediated translation (91). Only the full-length segment of the J-K region showed interaction with HEAT-1 protein, suggesting that the adjuster module accomplishes the preorganization of the J-K region. This pentaloop motif acts as a dual-sided docking station for base pair receptors. These mechanistic remodeling in the organization make the IRES of the EMCV more flexible to interact with those proteins that are impossible to dock (128). The 40S ribosomal subunit protein, RACK1, has modulated the IRES activity of some nonpicornaviruses. Inhibition of RACK1 significantly decreased the EMCV IRES activity; however, they did not show an interaction between RACK1 and EMCV IRES (57). In recent years, dead box family proteins have been reported to play major roles in viral inhibition. DDX60 was shown to inhibit FMDV and EMCV IRES-dependent translation and replication. DDX60 inhibited EMCV and FMDV replication via modulating ribosomes on *in vitro*-transcribed type II IRES and viral mRNAs (129). Type II IRES is the leading group of IRES types regarding more ITAF discoveries. Nevertheless, more insight studies and discoveries of ITAFs will help us understand the viral replication mechanism and restrict them appropriately.

### Herbal extracts, chemicals, and drugs influencing type II IRES activity and virus replication.

Besides host proteins, some synthetic or natural compounds also have a significant role in the IRES-dependent translation activity. The host cap-dependent translation is shut down during FMDV infection by cleaving eIF4G via FMDV L^pro^. The cleaved eIF4G is hijacked to facilitate the cap-independent translation of the virus. The cells treated with different doses of 20 μg/mL apigenin and transfected with pIRES-green fluorescent protein (GFP) showed suppressed translation of FMDV, indicating that apigenin hindered the IRES-driven translation (130). A recent study has shown that pycnogenol (a compound of natural chemicals from the bark of a fine European tree) was treated for 72 h in the B10 cell line expressing FMDV IRES, which showed a significant decrease in FMDV IRES activity. Additional results showed that 115 host genes were downregulated using pycnogenol, which suggested that the suppression of FMDV IRES activity could be due to the downregulation of host genes (131). Studies regarding herbal extracts summarized in this review, compared to type I IRES viruses, the herbal extracts that can potentially restrict IRES-dependent translation are negligible in type II IRES viruses, especially those against FMDV. Therefore, more studies are required on the discoveries of herbal extracts, which may help to contain type II IRES viruses.

## TYPE III IRES AND THE ITAFS REGULATING ITS TRANSLATION

### Type III IRES structure.

Hepatitis A virus IRES is 734 nt long. It contains six domains (I to VI). Domains I to II are composed of 1 to 95 nt, which include two stem-loops and a pyrimidine-rich tract. In contrast, domains III to VI comprise 155 to 734 nt, which comprise several complex stem-loops (132). The schematic diagram of the type III IRES is shown in Fig. 2C.

### Initiation factor requirement.

Hepatitis A virus IRES is very inefficient (133) and thus requires all host initiation factors to form a translation initiation complex, including eIF4E and eIF4G (134). However, many other picornaviruses do not require these translation initiation factors (38).

### Positively regulating ITAFs.

This type of IRES is also regulated by many ITAFs, which are discussed below. The HAV 5′ UTR showed competition for PCBP2 with the stem-loop IV of the PV RNA to promote viral IRES-driven translation. It was shown that H1-354 is the essential part of HAV IRES, which contains important pyrimidine-rich sequences and stem-loops. It showed less likely competition than the whole 5′ UTR, and further poor competition was observed when more small fragments were used, which could be due to the unstable structures compared to the full-length RNA. Furthermore, the first 157 nucleotides of H1-354, including the pyrimidine-rich tract, were the precise site of binding in HAV IRES for PCBP2 (135). PTB was reported to enhance HAV IRES activity more efficiently than poliovirus by interacting with domain IIIa of the HAV IRES (30, 31).

The cleavage of host protein through viral proteases is a common phenomenon to promote viral replication. A study by the Redondo group shows that the cleavage of eIF4G by FMDV-L^pro^ promoted HAV IRES translation while inhibited by poliovirus 2A^pro^. Further results showed that pFMDV L^pro^ resulted in cleavage of eIF4G and pronounced stimulation of HAV Luc synthesis. These observations were further confirmed in the Huh7-T7 cells, which showed that pFMDV-L^pro^ increases, while poliovirus 2A^pro^ decreases, HAV IRES luciferase mRNA synthesis. Altogether, this study suggests the opposite function of both proteases on HAV IRES, and the translation is carried out in the presence of phosphorylated eIF2a and cleaved eIF4G (136). A recent study showed that HAV IRES requires eIF4E to promote the IRES translation in a cell type-specific manner. Although 3′ UTR and poly(A) tail activity were not required for efficient HAV IRES translation in HeLa cells, they were necessary in Huh-7 cell extracts (134).

### Negatively regulating ITAFs.

Some ITAFs have been reported showing negative HAV IRES activity regulation. The 3C protease of HAV cleaved PABP into the C terminus and N terminus. The cleaved N terminus negatively regulated HAV IRES translation, while the C terminus had no effect. Furthermore, the N terminus showed an enhanced binding affinity for viral 5′ UTR pY1 to suppress its viral genome replication ability (137). Similarly, the nuclear resident protein La autoantigen binds with the cellular and viral RNA and works as a transactivating factor to modulate the translation. The La autoantigen binds to the HAV IRES in three regions. Interestingly, it binds to the region of domain IIIa competitively to glyceraldehyde-3-phosphate-dehydrogenase (GAPDH) and PTB. Surprisingly, when the native protein La autoantigen was added to the RRL, the PV IRES activity was enhanced; however, HAV IRES activity was suppressed. In addition, PV virus 3C^pro^ cleaved La autoantigen, but HAV 3C^pro^ did not, which suggested that the cleavage of La autoantigen was required for enhanced IRES activity (30).

Viral IRESs also benefit from protein-protein competition. GAPDH and PTB were reported to competitively interact with domain IIIa of the HAV IRES and show an antagonist effect on HAV IRES activity (30). The overexpression of GAPDH greatly suppressed the HAV IRES translation in FRhK-4 and BSC-1, but not in Huh-7 cells. Huh-7 cells have a higher polypyrimidine tract-binding protein (positive regulator of HAV IRES activity) in their cytoplasm, which neutralized the GAPDH-negative regulatory activity (138).

It was hypothesized that HAV does not interfere with host cellular translation shutoff, and a previous study showed that HAV proteinase 3C decreased IRES-driven translation via cleaving the HAV IRES-interacting protein PTB, which suggests that the HAV protein interferes with its RNA to promote its genome replication (139). The HAV IRES is unique among all the picornaviruses; its IRES translation is promoted by adding eIF4E while it was inhibited by 4E-BP1. However, no effect was observed on the IRES translation of classical swine fever virus (CSFV), EMCV, PV, or HRV. In contrast, the HAV IRES was significantly inhibited by this protein, showing its uniqueness from the other IRESs. When the eIF4E was added, the HAV IRES activity was reversed (38). The above studies showed that cleavage is the main tool of viruses to harness cellular proteins for their translation and replication. A couple of studies showed the interaction of HAV IRES and ITAFs; therefore, more insight studies are required to discover various ITAFs to understand the virus-host interaction better and find the remedies for viral infection.

### Herbal extracts, chemicals, and drugs influencing type III IRES activity and virus replication.

An antiviral agent, amantadine, and interferon alpha were used in different combinations to evaluate HAV IRES activity and HAV replicon replication. Amantadine was observed to suppress HAV IRES activity, albeit interferon alpha showed no additive effect on HAV IRES activity through amantadine. Furthermore, the combination of amantadine and interferon alpha showed stronger inhibition of HAV replicon and replication (140). Similarly, Kanda et al. showed that interleukin 29 (IL-29) significantly suppressed HAV IRES activity, and the costimulation of IFN-α, amantadine, and IL-29 showed much stronger suppression (141). Sirtinol, a sirtuin inhibitor, suppressed HAV IRES-driven translation in COS7 HAV IRES cells. Further results showed that 10 μM sirtuin caused a 67% decrease in HAV replication, and the same amount of inhibitor decreased HAV IRES activity to 83.6% (142). A recent study showed that Japanese rice-koji miso extract enhances glucose-regulated protein 78 (GRP78), a heat shock protein family member needed for stress-induced autophagy, and endoplasmic reticulum stress, which suppresses HAV replication (143). To our best knowledge, the above-described studies reported that it suppresses HAV replication. More detailed studies are warranted to determine the effects of herbal extracts or drugs on HAV IRES translation.

## TYPE IV IRES TRANSLATION REGULATION

### Type IV IRES structure.

Type IV IRESs exist in *Picornaviridae*, such as sapelovirus A, sapelovirus B, teschovirus A, and SVA, as well as members of the *Flaviviridae* family, e.g., CSFV and hepatitis C virus (HCV) (33, 41, 144).

Senecavirus A is a small, 27- to 30-nm, single-stranded positive-sense RNA virus and is the only virus in the genus *Senecavirus*. Its genome is approximately 7,280 nt long, encompassing the 5′-UTR 666 nt with a single ORF of 6,543 nt, followed by a 3′ UTR of 71 nt and a poly(A) tail, whereas SVA P1, 2C, 3C, and 3D are similar to that of cardioviruses (145). However, SVA IRES sequence was 52% identical to HCV and 47% identical to CSFV IRES (144). An inhibitor, hippuristanol, was used in HEK293T cells for eIF4A activity. SVA IRES activity shows significant resistance to this inhibitor, which illustrates that it differs from other *Picornaviridae* members, such as EMCV, which showed a marked decrease in FLuc activity and cap-dependent expression of CAT. These findings show that like HCV, SVA IRES also does not need eIF4A for its IRES activity (144). The sequence alignment results showed that SVA IRES is 47% similar to that of CSFV, while it is 52% identical to HCV IRES. However, these IRES share a sequence at particular dIIIa, dIIIc, IIIe, and a small motif within domains IIId1 and II (146, 147). The schematic diagram of type IV IRES is shown in Fig. 2D.

A recent study has confirmed that the point mutation induced in the pseudoknot stem Ib (PKS-Ib) of senecavirus A proved indispensable for viral growth (148). The subdomains IIId2 of border disease virus (BDV) and CSFV are essential for the IRES activity and 80S ribosome assembly. However, an additional IIId2 subdomain in SVA, similar to that of CSFV and BDV, has no role in IRES activity. Furthermore, the deletion of IIId2 affects SVA growth and RNA synthesis. In addition, IIId2 is not required to recruit eIF3 or 40S in BDV and CSFV; it has an essential role in 80S complex formation in the pestiviruses mentioned above (149). Future studies should focus on the SVA IRES organization and its role in viral replication.

## TYPE V IRES AND THE ITAFS REGULATING ITS TRANSLATION

### Type V IRES structure.

*Kobuvirus*, *Salivirus*, and *Oscivirus* have a distinct type of IRES known as IRES type V (42, 152). *Kobuvirus* comprises six species, *Aichivirus A*, *B*, *C*, *D*, *E*, and *F* (150). Aichi virus A has an 8,251-nt-long genome. The 5′ UTR is 712 nt, and the 3′ UTR is 240 nt long, followed by a single ORF and poly(A) tail (151). To properly replicate its viral RNA, Aichi virus has adopted a distinguished IRES known as type V IRES, composed of 8 domains or secondary elements, E, F, G, H, I, J, K, and L, downstream of domains A to D. The schematic diagram of the type V IRES is shown in Fig. 2E.

The domain I sequence is unique to the AV. The importance of the domains from A to J was assessed by deletion, and it was found that the IRES activity was abolished, while deletion of domains A to I decreased the activity of IRES by 90%. However, AV's domain I is distinct from type I, II, and III IRES viruses (152). Three stem-loops (SLs), known as SL-A, SL-B, and SL-C, were identified in its 5′ UTR. Mutagenesis of seven base pairs in the center part of the SL-A showed its significance for the plaque-forming activity (153). A novel sequence of a 43-nt-long stem-loop was identified at the 5′ UTR of AV. Mutagenesis at various sites showed its importance for viral RNA replication and virulence (154). The SL-B sequence was found to be very significant for viral RNA replication. However, mutations induced to the SL-C were of little significance, only affecting the viability of the virus (155). Altogether, these three SLs are highly significant for negative- and positive-strand RNA replication (156). Mutation induced in stem-loops A and B indicated that the interaction between 3ABC and these SLs was drastically impaired. However, the specific sequence of stem-loop B was significant for this interaction. The interaction between P3 and the three stem-loops mentioned above showed that they were critically required to synthesize negative-strand RNA (157).

Domain J is smaller but similar to domain VI of type I IRES, containing GNRA, which is essential for IRES activity. The apical portion of domain K in AV IRES is similar to the apical part of domain J in type II IRES, which is considered essential for the eIF4G interaction. Deleting the GNRA motif in domain J decreased AV IRES activity to 20%. Furthermore, the substitution of an individual base pair surprisingly increased or showed no effect on IRES activity, explaining that the upper tetraloop has different activity than types 1 and 2 IRESs, although interference of the eIF4G-interacting motif abolished the AV IRES activity, pointing to the resemblance to type II IRES. Moreover, it was shown that DHX29 is an essential element for the 48S complex formation, which can enhance the complex formation more efficiently than PTB (152). The eIFs used in canonical translation were insufficient to initiate the translation on AV (89). Few studies reported that ITAFs regulate type V IRES translation. Future studies should focus on the interaction of type V IRES and host proteins, specifically the ITAFs, which may regulate the translation initiation.

## ITAF mRNAS INCREASE OR DECREASE AND TRANSLOCATION IN RESPONSE TO INFECTION

**FIG 3:**
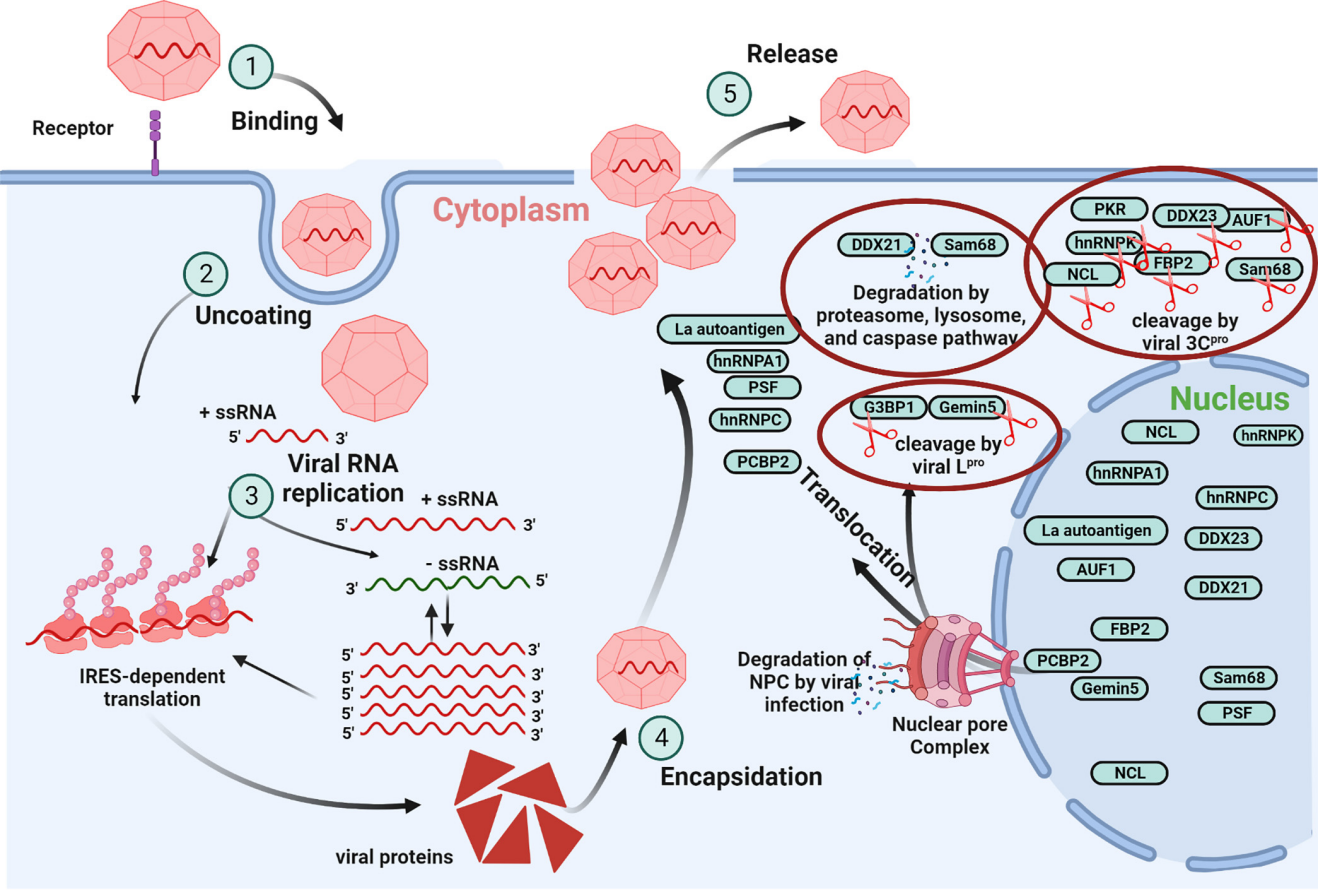
Picornavirus life cycle and ITAF translocation during the viral infection. The picornavirus life cycle has been shown in different stages, including binding, uncoating, viral RNA replication, encapsidation, and release. As a result of viral infection, the swarm of ITAFs can be seen moving from the nucleus to the viral replication sites in the cytoplasm. The localization of these ITAFs is the consequence of many mechanisms. The nuclear pore complex is degraded by viral infection to make it easy for ITAFs to enter the viral replication factories. Furthermore, viral proteins degrade the host proteins to promote their IRES-dependent translation.

One of the leading causes of cellular protein inflation is the cellular protein synthesis shutdown. These changes need hours to days, depending on protein stability, and they include mRNA inflation and shuttling of proteins. The role and mechanism of the nuclear pore complex (NPC) need to be explained to understand the shuttling of proteins from the nucleus to the cytoplasm. The NPC mainly controls nucleocytoplasmic transport, often hijacked by viral proteins to shuttle host nuclear proteins to the cytoplasm (158). The macromolecular assembly of NPC comprises around 110 MDa in vertebrates and 60 MDa in yeast. NPCs have an octahedral orientation across the two folds of the nuclear envelope, similar to bicycle wheel spokes. They have specialized linking proteins called nucleoporins, or Nups (159). In cells infected with the Sindbis virus (SINV), at 18 h postinfection (hpi), significant upregulation and downregulation of mRNAs were observed in response to viral infection. Although at 18 hpi, most mRNAs were viral RNA, which makes drastic changes in the influx or outflux of RBPs, dormant RBPs could be awakened by recognizing signatures within the viral RNA (160). According to Vladimer et al., all RBPs prevent viral replication but do not need to move into the cytoplasm. However, a few antiviral RBPs show this movement (161). Following the footsteps of the mRNA upregulation strategy, PKR mRNA was significantly increased; however, in response, the virus degrades its protein expression, and thus, the battle continues until one succeeds (162). Altogether, these proteins combat the virus by increasing their mRNA level to neutralize the viral activity. Many factors stimulate the trafficking of proteins between the nucleus and cytoplasm. Below, we will discuss the host nuclear proteins translocated from the nucleus to the cytoplasm during viral infections, as shown in Fig. 3.

Some proteins’ expression and mRNA levels are increased when the cells are infected with a virus, such as infecting HeLa S3 cells with CVB3 indicated that the PTB-associated splicing factor (PSF) protein expression and mRNA level were increased compared to mock-infected cells. Also, the phosphorylation of PSF promoted its shuttling from the nucleus to the cytoplasm. In addition, it also enhances the RNA-binding activity of PSF. In agreement with the above, phospho-dead mutants of PSF did not localize to the cytoplasm in mock and infected cells. The increase in protein expression level is due to the presence of IRES in PSF mRNA, which helps to survive and replicate its mRNA during viral infection and canonical protein synthesis shutoff (70, 163, 164).

FBP2 is a negative regulator of the EV-A71 virus replication, which shows translocation to the cytoplasm during viral infection. There could be two scenarios for this localization; first, FBP2 may have some important functions in the cytoplasm, and second, EV-A71 infection may have caused the localization of this protein (72). Viruses use counterdefense mechanisms against those proteins that subvert their poisonousness and hunt them down. These mechanisms include team working of viral proteins to hijack host proteins, such as poliovirus protease 3CD cleaving AUF1 and viral proteinase 2A shuttling AUF1 from the nucleus to the cytoplasm. Poliovirus 2A lacking proteinase activity halted the migration of AUF1 from the nucleus to the cytoplasm, which indicated the migratory function of 2A proteinase (165). Another mechanism is to halt the transport of the nuclear import pathway. Previously, it was considered that the cytoplasmic relocalization of proteins was due to the viral-induced inhibition of cellular transcription (166); however, when actinomycin D was used to inhibit the cellular transcription, it was found that the relocalization was more dramatic in infected cells than those treated with actinomycin D, which confirms that cellular transcription inhibition is not the sole player in this saga (167). Besides the mechanisms mentioned above, a short 40-amino-acid-long motif termed KNS mediates hnRNP K movement to the cytoplasm during viral infection (168). The proteins mentioned above are shuttling proteins; they move between the nucleus and cytoplasm. However, some nonshuttling proteins also show this movement, such as hnRNP C, which relocalized to the cytoplasm with poliovirus infection to promote viral replication (169). However, the kinetics is much slower than the shuttling proteins (167).

Similarly, the nuclear protein Sam68 translocated to the cytoplasm with EV-A71, FMDV, and bovine enterovirus 1 (BEV1) infection, where it was associated with PABP and PCBP2 (170). The mechanism of FMDV 3C^pro^-dependent translocation of Sam68 is explained below. Sam68 was cleaved by FMDV 3C^pro^ at the C terminus, encompassing the nuclear localization sequence. The size of the cleaved portion was less than 50 kDa, which can easily move through the nuclear pore complex, and thus, Sam68 translocates to the FMDV replication factories in the cytoplasm. Furthermore, Sam68 interacted with the IRES of the FMDV, which compelled this protein to the cytoplasm. In addition, Nups 153, 62, and 98 were not degraded in the early stages of Sam68 translocation to the cytoplasm during FMDV infection (104). In contrast, Nup 98 and Nup 62 were quickly degraded during rhinovirus and poliovirus infection; however, Nup 153 degradation was very slow (171). Furthermore, the cleavage of Nup proteins was reported in various kinds of virus infections, such as NS2B3 protease of dengue virus, and Zika virus (ZIKV) was responsible for the cleavage of Nup62, 98, and 153. Dengue virus infection caused a reduction of 31% in Nup62 expression; however, no reduction was observed with ZIKV, whereas in the case of Nup98 and Nup153, a dramatic reduction was observed in both dengue virus- and ZIKV-infected cells, and disruption of the nuclear pore was also observed, as shown in Fig. 3 (158). Similarly, Nup98 was cleaved by CVB3 2A, which subsequently promoted myocarditis by impairing the NRG1-ERBB4/PSEN1 signaling cascade (172).

Gemin5 translocated to the cytoplasm and interacted with FMDV IRES (111). Similarly, Gemin5 was strongly stimulated by SINV and localized to the viral replication factories. Surprisingly, the other partners of Gemin5 were not activated by SINV (160). HnRNP A1 is a nuclear protein that shuttles between the nucleus and cytoplasm. EV-A71 virus infection causes hnRNP A1 to translocate to the cytoplasm. However, in the case of SINV infection, hnRNP A1 was not localized to the cytoplasm. These observations provide further insight into the translocation of proteins from the nucleus to the cytoplasm (160).

In contrast, Lin et al. reported that hnRNP A1 was localized to the cytoplasm during SINV infection (173). However, EV-A71 proteases 3A and 3C did not independently induce localization of hnRNP A1, suggesting that other viral proteins collectively compel hnRNP A1 to the cytoplasm to facilitate viral replication (173). Furthermore, this phenomenon was also observed in HCV and mouse hepatitis virus, which also require hnRNP A1 as an important ITAF for their replication (174, 175). Cytoplasmic localization during EV-A71 viral infection of hnRNP A1 is crucial for viral IRES to recruit the 40S ribosomal subunit (65). Similarly, the nuclear protein NCL showed translocation to the cytoplasm to interact with FMDV IRES during FMDV infection. In addition, it showed localization with FMDV 3C^pro^ (108). Viruses often cleave host proteins to promote their replication or benefit from them. SVA 3C^pro^ cleaved host nuclear protein NCL during infection, which caused its translocation to the cytoplasm and promoted viral replication (176). However, in the case of FMDV infection, NCL does not show any cleavage. In conclusion, viruses use multiple ways to hijack host proteins to enhance their replication. Future studies should focus on finding out the in-depth mechanism of the translocation of host proteins during viral infection from the nucleus to the cytoplasm to better understand the virus replication mechanism.

### Conclusion.

The increasing number of viral species with pace of time indicated the increase in types of IRESs discovered in picornaviruses. IRESs have diverse functions and various requirements of eIFs and ITAFs. An increase in the discoveries of novel ITAFs has been reported, which is progressively moving forward. The discoveries of ITAFs substantially contributed to understanding viral replication behavior that helped us restrain viruses more effectively than ever before. Herbal extracts, drugs, and chemicals used against some viruses have proven to be versatile for restraining viruses, although the use of these agents is only restricted to type I, II, and III IRESs. There is an urgent need for further research on such kinds of natural or synthetic agents to control viruses effectively. The trafficking of the proteins discussed in this review and beyond should be deeply investigated to discover more information that will enhance knowledge about viral replication and their role in infection and cellular processes. As this review explained some significant points of protein trafficking, there is an urgent need to discover more about this phenomenon for a better understanding of their protein contributions and to benefit from it by making new control strategies for viruses.
